# Local radiotherapy and E7 RNA-LPX vaccination show enhanced therapeutic efficacy in preclinical models of HPV16^+^ cancer

**DOI:** 10.1007/s00262-021-03134-9

**Published:** 2021-12-31

**Authors:** Nadja Salomon, Abderaouf Selmi, Christian Grunwitz, Anthony Kong, Eliana Stanganello, Jennifer Neumaier, Jutta Petschenka, Mustafa Diken, Sebastian Kreiter, Özlem Türeci, Ugur Sahin, Fulvia Vascotto

**Affiliations:** 1 TRON - Translational Oncology at the University Medical Center of the Johannes Gutenberg University gGmbH, Freiligrathstraße 12, 55131 Mainz, Germany; 2 Biopharmaceutical New Technologies (BioNTech) SE, Mainz, Germany; 3 Comprehensive Cancer Centre, King’s College London, London, UK; 4 Research Center for Immunotherapy (FZI), University Medical Center at the Johannes Gutenberg University, Mainz, Germany; 5 Present Address: Boehringer Ingelheim Pharma GmbH & Co. KG, Biberach an der Riss, Germany

**Keywords:** HPV16 E6/E7 cancer, RNA vaccines, Local radiotherapy, Anti-tumoral CD8^+^*T *cells, Tumor environment

## Abstract

Human papilloma virus (HPV) infection is a causative agent for several cancers types (genital, anal and head and neck region). The HPV E6 and E7 proteins are oncogenic drivers and thus are ideal candidates for therapeutic vaccination. We recently reported that a novel ribonucleic acid lipoplex (RNA-LPX)-based HPV16 vaccine, E7 RNA-LPX, mediates regression of mouse HPV16^+^ tumors and establishes protective *T *cell memory. An HPV16 E6/E7 RNA-LPX vaccine is currently being investigated in two phase I and II clinical trials in various HPV-driven cancer types; however, it remains a high unmet medical need for treatments for patients with radiosensitive HPV16^+^ tumors. Therefore, we set out to investigate the therapeutic efficacy of E7 RNA-LPX vaccine combined with standard-of-care local radiotherapy (LRT). We demonstrate that E7 RNA-LPX synergizes with LRT in HPV16^+^ mouse tumors, with potent therapeutic effects exceeding those of either monotherapy. Mode of action studies revealed that the E7 RNA-LPX vaccine induced high numbers of intratumoral-E7-specific CD8^+^ *T *cells, rendering cold tumors immunologically hot, whereas LRT primarily acted as a cytotoxic therapy, reducing tumor mass and intratumor hypoxia by predisposing tumor cells to antigen-specific *T *cell-mediated killing. Overall, LRT enhanced the effector function of E7 RNA-LPX-primed *T *cell responses. The therapeutic synergy was dependent on total radiation dose, rather than radiation dose-fractionation. Together, these results show that LRT synergizes with E7 RNA-LPX and enhances its anti-tumor activity against HPV16^+^ cancer models. This work paves into a new translational therapy for HPV16^+^ cancer patients.

**Supplementary Information:**

The online version contains supplementary material available at 10.1007/s00262-021-03134-9.

## Introduction

HPV is the most frequently sexually transmitted viral infection [1] and, owing to the expression of the viral oncogenes E6 and E7, is an essential driver in the induction of genital, anogenital and head and neck cancers [2]. A total of 100% cervical, 88% anal, 78% vaginal and 31% oropharyngeal cancers are attributed to infection with the oncogenic viruses HPV16 and HPV18 [3]. In the head and neck cancer region, mainly the oropharynx, HPV16 is the most common oncogenic virus, with 85% of oropharyngeal head and neck squamous cell carcinomas (HNSCCs) being HPV16^+^. Independent of HPV status, HNSCCs are classically treated by surgery, LRT, platinum-based chemotherapy or a combination, depending on the stage and location of tumors [4]. HPV^+^ HNSCCs generally have a more favorable prognosis [5] and, due to the deregulation of the cell cycle and deoxyribonucleic acid (DNA) repair by the oncoproteins E6 and E7, are more radiosensitive than their radioresistant HPV^−^ counterpart [6] as they accumulate unrepaired double-strand breaks and undergo cell cycle arrest G2 [7].

In addition to their contribution to radiosensitivity, the oncoproteins E6 and E7 are exclusively expressed in tumor tissue and are highly foreign to the immune system, rendering them ideal candidates for therapeutic anti-cancer vaccination. Several therapeutic HPV vaccines are currently being investigated in clinical trials [8], *e.g.* for *Listeria monocytogenes-*based (*Lm*-LLO-E7) [9], DNA-based (VGX-3100) [10] or virus-based [11] HPV vaccines. Whereas bacterial- and viral-based vaccines face complex manufacturing steps and potentially immunity against vectors upon re-immunization, DNA-based vaccines possess the danger of stable integration. As a vaccine format, RNA is non-integrative, inherently immunogenic, possesses the advantage to target several lymphoid organs with a high reservoir of antigen presenting cells (APC), and via in vitro transcription cost-effective to manufacture [12]. We recently reported the efficacy of a novel systemic RNA-based HPV16 vaccine, E7 RNA-LPX, that mediates the regression of well-established HPV16^+^ mouse tumor models TC-1 and C3 [13]. RNA-LPX-based vaccines are based on charge-optimized, liposomal-formulated, single-strand antigen-encoding RNA that allow the systemic delivery of antigens to antigen-presenting cells in lymphoid organs after intravenous injection [14]. Antigen-encoding RNA is engineered for optimized intracellular stability and translational efficiency [15, 16] and for epitope presentation on MHC class I and MHC class II molecules [17]. The E7 RNA-LPX vaccine primed high numbers of cytotoxic E7_49–57_-specific CD8^+^ *T *cells that infiltrated s.c. and mucosal TC-1 and C3 tumors, drove tumor rejection and formed potent memory [13]. In HPV16^+^ mouse tumor models, regression was nevertheless followed by tumor relapse in some cases, especially when vaccination was initiated in mice with long-established tumors. Similar observations were made by others using HPV16 DNA [18, 19] or peptide-based [20–22] vaccines.

The safety and therapeutic efficacy of an HPV16 E6/E7 RNA-LPX vaccine is currently being investigated in a phase I clinical trial in patients with HPV-driven cancers including HNSCC, anogenital, cervical and penile cancers (NCT03418480) and in a phase II clinical trial in combination with pembrolizumab in patients with HPV16^+^ and PD-L1^+^ HNSCC (NCT04534205).

Given the limitations of single-agent therapy, combination therapy may provide advantages in terms of tumor response and relapse rates, particularly in advanced stage cancers. As HPV-driven cancers are particularly radiosensitive and as LRT is a standard-of-care modality in patients with cervical and head-neck cancer, we investigated the therapeutic efficacy of LRT and RNA-LPX-based HPV16 E7 vaccines for future clinical translation. We used the well-established HPV16^+^ tumor models TC-1 and C3 to investigate the impact of E7 RNA-LPX vaccines combined with different doses of LRT on survival, antigen-specific immune responses, frequency and phenotype of tumor immune infiltrates as well as of tumor cells.

## Materials and methods

### Mice

Female C57BL/6 wild-type mice (8 to 10 weeks) were purchased from Envigo.

### Tumor cell lines

The mouse HPV16 E6/E7^+^ TC-1 tumor cell line [23] and TC-1 luciferase variant were obtained from T.C. Wu (Johns Hopkins University). The mouse HPV16^+^ C3 tumor cell line [20] was a kind gift by S.H. van der Burg (Leiden University Medical Center). Tumor cells were irradiated with the orthovoltage X-ray source X-RAD320 (Precision X-Ray Inc.) at a dose rate of 0.93 Gy/min.

### *RNA constructs and *in vitro* transcription*

Plasmid templates for in vitro transcription of antigen-coding RNAs were generated, cloning E7 (encoding full-length HPV16 E7) or chicken ovalbumin (OVA, encoding the H-2 Kb-epitope OVA_257-264_) target sequences for fusion with major histocompatibility complex (MHC) class I transmembrane and cytoplasmic domain [13–15, 17]. Antigen-encoding vectors were in vitro transcribed and capped with the β-S anti-reverse cap analog [16]. OVA RNA was used as a control RNA.

### RNA-LPX preparation

RNA-LPX were generated by complexing negatively charged RNA with cationic liposomes at a ( +):(-) charge ratio of 1.3:2, as previously described [14]. RNA-LPX had a particle size of 200–250 nm, a polydispersity index of ~ 0.25 and a zeta potential (mV) of -20–30 mV.

### Tumor models and treatment

C57BL/6 mice were injected with 1 × 10^5^ TC-1 or TC-1 luc tumor cells and 5 × 10^5^ C3 tumor cells subcutaneous (s.c.) into to the right flank. Tumor growth was measured unblinded with a caliper and tumor volumes calculated by (a^2^ × b)/2 (a, width; b, length). Mice were randomized according to tumor size and immunized intravenously with 40 µg E7 or control (OVA) RNA-LPX. The orthovoltage X-ray source X-RAD320 (Precision X-Ray Inc.) was used for tumor irradiation locally with either 12 Gy or a similar biologically effective dose (BED) of 3 × 6 Gy at a dose rate of 0.47 Gy/min. BEDs were calculated using the formula $BED=\mathrm{n}\times \mathrm{d}(1+\frac{\mathrm{d}}{\frac{\mathrm{\alpha }}{\upbeta }})$ with n being the number of fractions, d being the dose per fraction and $\frac{\mathrm{\alpha }}{\upbeta }$ being the tumors intrinsic radiosensitivity. [24] Tumor irradiation was performed under ketamine/xylazine (12 Gy) or isoflurane (3 × 6 Gy) narcosis as previously described [25]. For in vivo bromodeoxyuridine (BrdU) labeling, the BrdU base analog (BrdU Flow Kit, BD Bioscience) was injected intraperitoneal at 1 mg/mouse, 24 h prior to organ excision. The hypoxia probe pimonidazole (Hypoxyprobe Inc.) was injected intravenously at 1.2 mg/mouse 1 h prior organ excision.

### Tissue preparation

Single-cell suspensions were generated from digested tumors using the mouse tumor dissociation kit and gentleMACS™ dissociator (both Miltenyi Biotec) or from lymph nodes as previously described [13].

### Flow cytometry

Flow cytometry staining was performed on blood, tumor, tumor-draining lymph node and spleen single-cell suspensions. Cells were stained with viability dyes (eBioscience) according to manufacturer’s instructions. For extracellular staining, anti-mouse CD45, CD8α, CD4, CD44, NK1.1, programmed death ligand 1 (PD-L1), cleaved caspase-3 (CC3), Qa-1b, CD11b, CD86, CD103 (BD Pharmingen), programmed death 1 (PD-1), NKG2AB (Invitrogen), *T *cell immunoglobulin and mucin domain-3 (TIM-3), Fas, H-2 (pan MHC class I), F4/80, CD11c, Gr-1, XCR-1, pan MHC class II (BioLegend) and CD25 (eBioscience) antibodies were used. For intracellular staining, anti-mouse interferon γ (IFNγ), interleukin-2 (IL-2, eBioscience) and tumor necrosis factor α (TNFα), Foxp3 (BD Pharmingen) antibodies were used. E7-specific CD8^+^ *T *cells were stained with E7_49–57_ H2-Db-restricted dextramers (Immudex). For IFNγ, TNFα and IL-2 staining, samples were fixed and permeabilized with Cytofix/Cytoperm (BD Pharmingen). For Foxp3, staining was carried out with the Foxp3 Fixation Kit (eBioscience) according to manufacturer’s instructions. Intracellular cytokine staining was performed as described earlier [26], stimulating CD8^+^ *T *cells with 2 µg/mL E7_49–57_ (RAHYNIVTF, Jerini Peptide Technologies) peptide-loaded C57BL/6 bone marrow-derived dendritic cells (BMDC) in the presence of 10 µg/mL Brefeldin A (Sigma) for 5 h at 37 °C. Peripheral blood was stained with MHC-dextramers as previously described [13]. In vivo BrdU labeling and staining was performed using the in vivo BrdU flow cytometry kit (BD Pharmingen) according to manufacturer’s instructions. Immune cell populations were defined by pre-gating on viable cells and singlets and determined as follows: natural killer (NK) cells (CD45^+^ NK1.1^+^), CD8^+^ *T *cells (CD45^+^ CD8^+^), E7-specific CD8^+^ *T *cells (CD45^+^ CD8^+^ E7_49-57_ multimer^+^), CD4^+^ *T *cells (CD45^+^ CD4^+^), regulatory *T *cells (Treg, CD45^+^ CD4^+^ Foxp3^+^ CD25^+^), tumor cells (CD45^−^ CD44^+^), tumor associated macrophages (TAM), (CD45^+^ F4/80^+^ CD11b^+^), M1 TAM (CD45^+^ F4-80^+^ CD206^−^ MHC class II^+^), M2 TAM (CD45^+^ F4/80^+^ CD206^+^), myeloid suppressor cells (MDSC) (CD45^+^ F4/80^−^ Gr-1^+^ CD11b^+^), type I DC (DC1) (CD45^+^ F4/80^−^ Gr-1^−^ CD11c^+^ CD11b^−^ XCR1^+^ CD103^+^), type 2 DC (DC2) (CD45^+^ F4/80^−^ Gr-1^−^ CD11c^+^ CD11b^+^). Flow cytometric data was acquired on FACS Canto II or LSR Fortessa (BD Biosciences) and analyzed using FlowJo 10.4 (Tree Star).

### Immunofluorescence microscopy

8 µm sections of cryoconserved tumors were fixed in 4% paraformaldehyde for 10 min, permeabilized in tris-buffered saline containing 0.1% triton and blocked in Dulbecco's phosphate buffered saline supplemented with 1% bovine serum albumin, 5% mouse serum, 5% rat serum and 0.1% triton for 1 h. Fluorescently labeled antibodies against pimonidazole-thiol adducts (Hydroxyprobe™ Red584, Hydroxyprobe Inc.), TOX1 (Miltenyi Biotec), CD8^+^ (BD Pharmingen) were used to stain sections overnight at 4 °C, followed by nuclear staining with Hoechst (Thermo Fisher Scientific). Immunofluorescence images were acquired using the tile scanning function of the epifluorescence microscope (Axio Scan.Z1, Zeiss). Images of four tumor layers (n = 4 mice/group) were analyzed for quantification of pimonidazole positive areas among each tumor section by an automatized script available in Fuji/ImageJ software. Images of two tumor layers (n = 3 mice/group) were analyzed for TOX1 quantification using the image analysis algorithm (HighPlex FL v3.1.0) of the HALO® software (HALO v3.0.311.228, Indica Labs). Frequency of TOX1^+^ CD8^+^ *T *cells were quantified among Hoechst^+^ CD8^+^ *T *cells.

### Statistical analyses and data presentation

Data are presented as mean ± standard error of the mean (SEM). Statistical analysis was performed with GraphPad PRISM 8 and considered statistical significant when *p ≤ 0.05, **p ≤ 0.01, ***p ≤ 0.001. Unpaired, two-tailed Student’s t-test was used to compare single treatment and control group means. One-way analysis of variance (ANOVA) was performed if more than two experimental groups were compared, and when determined significant (p < 0.05), Tukey’s or Dunnett’s multiple comparison tests were used. Log-rank test (Mantel-Cox) was used to determine survival benefits.

## Results

### ***E7 RNA-LPX vaccination and LRT synergize to control established HPV16***^+^***tumors***

**Fig. 1:**
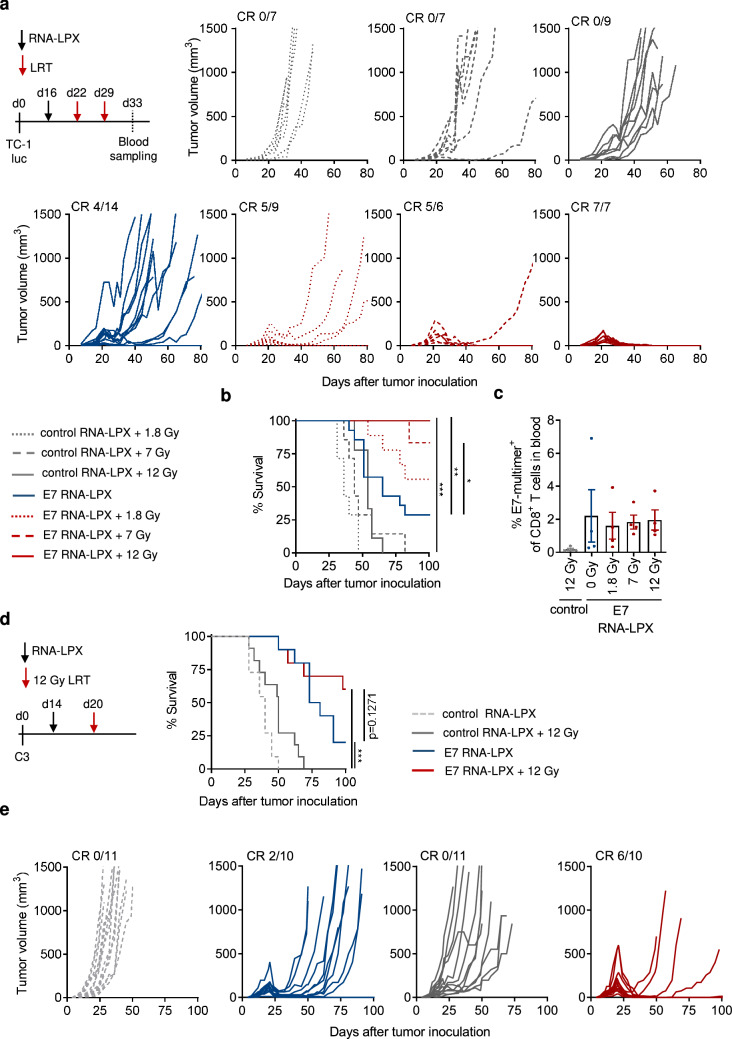
E7 RNA-LPX vaccination and LRT synergize to control established HPV16^+^ tumors. **a**–**c** TC-1 luc tumor-bearing C57BL/6 mice (*n *= 6–14/group) were vaccinated with E7 or control (OVA) RNA-LPX at a mean volume of 75 mm^3^ and subsequently locally irradiated with two weekly doses of 12 Gy, 7 Gy or 1.8 Gy. **a** Tumor volume and **b** survival were monitored over time. **c** The fraction of E7-multimer^+^ CD8^+^ T cells in the blood (*n* = 4/group) was determined by flow cytometry. **d**, **e** C3 tumor-bearing C57BL/6 mice (*n* = 10–11/group) were immunized E7 or control (OVA) RNA-LPX when tumors had reached a mean volume of 65 mm^3^ and subsequently locally irradiated with 12 Gy. **d** Survival and **e** tumor volume were monitored over time. Data are shown as mean ± SEM. Significance was determined using **b**, **d** Mantel-Cox log-rank test and **c** one-way ANOVA and Tukey’s multiple comparison test with *p ≤ 0.05, **p ≤ 0.01, ***p ≤ 0.001. CR: complete response; LPX: lipoplex; LRT: local radiotherapy

In order to evaluate the combination of E7 RNA-LPX and LRT, we designed a schedule employing a subtherapeutic dose of E7 RNA-LPX (late single intravenous immunization) followed by different doses of LRT (two weekly doses of 1.8 Gy, 7 Gy or 12 Gy) in mice bearing well-established (therapy start at 75 mm^3^) HPV16 E6/E7^+^ TC-1 tumors. In line with our previous findings [13] single and late-administered E7 RNA-LPX vaccination significantly promoted tumor rejection (Fig. 1a) and survival in TC-1 tumor-bearing mice compared to control-vaccinated mice (Fig. 1b); however, tumor rejection was followed by cases of relapse (10/14 mice). Double LRT treatment with control RNA-LPX only had a marginal effect on tumor growth compared to non-irradiated mice (control RNA-LPX) but, when combined with E7 RNA-LPX, displayed superior tumor rejection, independently of the tested radiation doses (Fig. 1a, b). Greatest anti-tumor efficacy was achieved when E7 RNA-LPX was combined with double treatment of high-dose LRT (12 Gy), rendering 100% of mice tumor-free up to 100 days after tumor inoculation (Fig. 1b) – a schedule that was chosen for subsequent experiments, however, dispensing the second irradiation to allow the collection of samples for characterization of the tumor immunemicroenvironment by flow cytometry. Seventeen days after E7 RNA-LPX vaccination, 2% of circulating CD8^+^ *T *cells were E7-specific whether mice were irradiated or not, indicating the efficient priming of antigen-specific *T *cell responses after E7 RNA-LPX with or without cytotoxic LRT (Fig. 1c).

Recent reports from studies in mice have shown that radiation dose-fractionation to an intermediate high-dose has superior *T *cell priming capacity when administered together with an anti-cytotoxic T-lymphocyte-associated protein 4 monoclonal antibody [27]. Therefore, we combined E7 RNA-LPX with 12 Gy fractionated to a similar BED of 3 × 6 Gy (single administration). The rate of survival was at 25% whether the E7 RNA-LPX vaccine was combined with 12 Gy or 3 × 6 Gy LRT, indicating the relevance of total dose rather than LRT dose-fractionation to reach therapeutic synergism with E7 RNA-LPX (Supplementary Fig. 1a, b). The frequency of E7-specific CD8^+^ *T *cells in E7 RNA-LPX- and E7 RNA-LPX/LRT-treated mice was monitored over time and displayed a peak around 12 days after vaccination (Supplementary Fig. 1c). Interestingly, E7 RNA-LPX/LRT-treated mice showed the highest persistence of E7-specific CD8^+^ *T *cells in the circulation, potentially accounting for prolonged and still ongoing tumor rejection in this treatment group.

The anti-tumor efficacy of combined E7 RNA-LPX/LRT was also evaluated in a second HPV16^+^ mouse tumor model, C3. In line with findings made in the TC-1 tumor model, the combination of E7 RNA-LPX vaccination and high-dose LRT (reduced to a single treatment) enhanced the survival benefit (Fig. 1d) and the rate of complete responses (CR 6/10 mice; Fig. 1e) of C3 tumor-bearing mice over monotherapies and control-treated mice.

### E7 RNA-LPX vaccination alone or in combination with LRT induces high levels of effector immune cell infiltration

The wide clinical use of radiotherapy is based on its cytotoxic and growth inhibitory properties; however, evidence in the last two decades suggests that radiotherapy may also activate the immune system, especially when given at a high-dose and combined with immunotherapy [28].

**Fig. 2:**
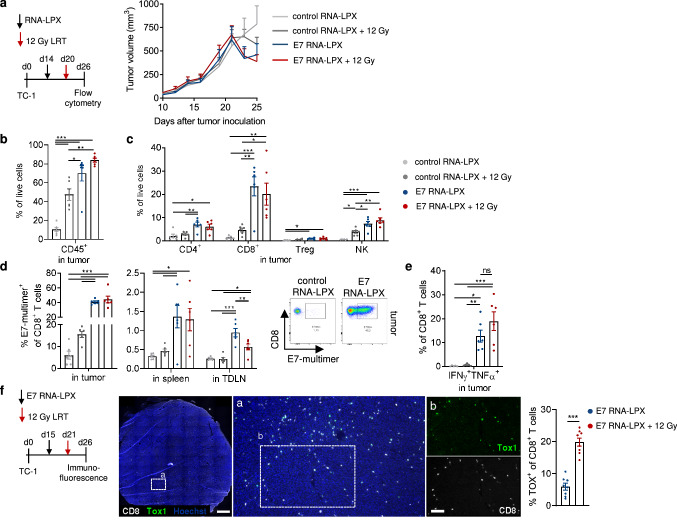
E7 RNA-LPX vaccination alone or in combination with LRT induces high levels of effector immune cell infiltration. **a** TC-1 tumor-bearing C57BL/6 mice (*n* = 6/group) were vaccinated with E7 or control (OVA) RNA-LPX at a mean volume of 165 mm^3^ and subsequently locally irradiated with 12 Gy. Tumor volume monitored over time. **b**–**d** Cellular composition evaluated by flow cytometry after excision of tumors, spleens and tumor-draining lymph nodes (inguinal) six days after irradiation. The percentages of tumor-infiltrating **b** CD45^+^ leukocytes, **c** CD4^+^, CD8^+^, Treg, NK cells and **d** fraction of E7-multimer^+^ CD8^+^ T cells in tumor, spleen and tumor-draining lymph nodes are shown. Representative pseudocolor plots show E7_49–57_ multimer staining in TC-1 tumors. **e** CD8^+^ TIL were enriched and restimulated ex vivo with E7_49–57_ peptide-loaded C57BL/6 and IFNγ and TNFα effector cytokines were detected by intracellular cytokine staining. **f**. TC-1 tumor-bearing C57BL/6 mice (*n* = 4/group) were immunized with E7 RNA-LPX at a mean tumor volume of 160 mm^3^ and subsequently locally irradiated with 12 Gy. Tumors were excised and stained for detection of TOX1 in CD8^+^ T cells. Fraction of TOX1^+^CD8^+^ T cells among CD8^+^ T cells is indicated. Representative images of CD8^+^ (white), TOX1 (green) staining in a TC-1 tumor (Hoechst nuclear staining, blue). Scale bars 1 mm (left) and 50 µm (right). Data are shown as mean ± SEM. Significance was determined using (**b**–**e**) one-way ANOVA and Tukey’s multiple comparison test (**f**) unpaired, two-tailed Student’s t-test with *p ≤ 0.05, **p ≤ 0.01, ***p ≤ 0.001. IFNγ: Interferon γ; LPX: lipoplex; LRT: local radiotherapy; NK: natural killer; TDLN: tumor draining lymph node; TNFα: tumor necrosis factor α; Treg: regulatory T cell

To characterize the underlying cellular drivers of tumor rejection after combined E7 RNA-LPX/LRT treatment, we analyzed TC-1 tumor immune infiltrates by flow cytometry (Fig. 2). TC-1 tumors were excised twelve days after a single E7 RNA-LPX vaccination and five days after single dose 12 Gy LRT, which was a point at which tumor growth curves of single therapies already diverged (Fig. 2a). E7 RNA-LPX vaccination significantly increased the infiltration of CD45^+^ leukocytes from 10% at baseline (control RNA-LPX) to 70%, which was also observed for E7 RNA-LPX/LRT-treated mice (Fig. 2b). LRT with control RNA-LPX also mediated CD45^+^ leukocyte infiltration compared to non-irradiated mice (control RNA-LPX-treated), although this was to a lesser extent than the E7 RNA-LPX vaccine. Furthermore, E7 RNA-LPX vaccinated mice displayed a higher fraction of intratumoral CD4^+^, CD8^+^, NK cells (Fig. 2c) and E7-specific CD8^+^ *T *cells in the tumor as well as in spleens and lymph nodes of treated mice (Fig. 2d) when compared to LRT with control RNA-LPX, which only mildly modulated these cell populations. In sum, total immune infiltrates of E7 RNA-LPX/LRT-treated mice largely recapitulated those of E7 RNA-LPX-vaccinated mice (Fig. 2b-d). Within the CD8^+^ *T *cell population, 15% of CD8^+^ *T *cells from E7 RNA-LPX and E7 RNA-LPX/LRT-treated mice expressed IFNγ and TNFα after ex vivo peptide restimulation (Fig. 2e), indicating comparable effector function of E7-specific *T *cells in both treatment groups. Interestingly, we detected a higher frequency of the transcription factor TOX1 (Fig. 2f) in intratumoral CD8^+^ *T *cells by histology, which is required for a sustained *T *cell effector function in cancer and chronic viral infection [29, 30].

Tumor-infiltrating myeloid cells were also analyzed (Supplementary Fig. 2). E7 RNA-LPX/LRT-treated TC-1 tumor-bearing mice display an enrichment of total CD11b^+^ myeloid cells, such as type 2 DC (DC2), M1-polarized inflammatory tumor associated macrophages (TAM) (expressing MHC class II, while negative for CD206), myeloid derived suppressor cells (MDSC) (Supplementary Fig. 2c) and also type 1 dendritic cells (DC1)﻿. The expression of the activation markers CD86 and MHC class II (Supplementary Fig. 2d, e) was similar across different myeloid cell subsets independent of the treatment performed, whereas PD-L1 expression was enhanced on myeloid cells of vaccinated mice (Supplementary Fig. 2f).

### Combining LRT with E7 RNA-LPX vaccination enhances tumor cell death, reduces hypoxia and promotes CD8^+^*T *cell proliferation

As there was no evidence of cytokine modulation in intratumoral E7-specific CD8^+^ *T *cells that explained the superior therapeutic efficacy of E7 RNA-LPX/LRT over E7 RNA-LPX monotherapy, we investigated the impact of treatment on tumor cells, which are the vital cellular subset directly targeted by antigen-specific *T *cells.

**Fig. 3:**
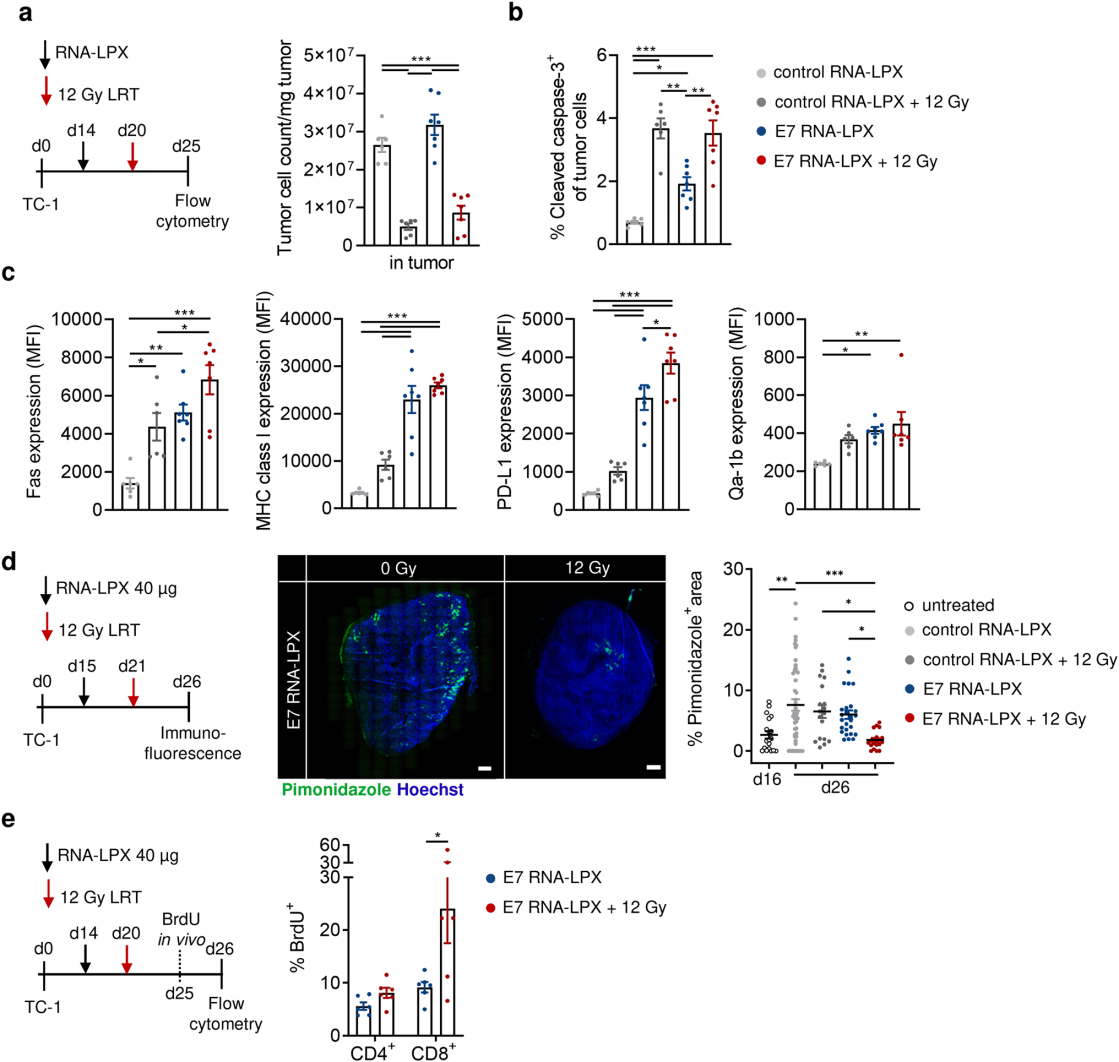
Combining LRT with E7 RNA-LPX vaccination enhances tumor cell death, reduces hypoxia and promotes CD8^+^ T cell proliferation. **a**–**c** TC-1 tumor-bearing C57BL/6 mice (*n* = 6–7/group) were vaccinated with E7 or control (OVA) RNA-LPX at mean volume of 125 mm^3^ and subsequently locally irradiated with 12 Gy. Five days after irradiation, tumors were excised for flow cytometry analysis. **a** TC-1 tumor cell count per mg tumor tissue. **b** Fraction of CC3^+^ TC-1 tumor cells and **c** expression of Fas, MHC class I molecules (pan H-2), PD-L1 and Qa-1b on TC-1 tumor cells. **d** TC-1 tumor-bearing C57BL/6 mice (*n* = 4/group) were immunized with E7 or control (OVA) RNA-LPX at a mean tumor volume of 160 mm^3^ and locally irradiated with 12 Gy. On day 16 (untreated) or on day 26 (five days after irradiation), the hypoxia probe pimonidazole was injected one hour prior to tumor excision. Tumor sections (*n* = 5/tumor) were stained by immunofluorescence. Hypoxic areas were quantified as pimonidazole^+^ area as a fraction of the total tumor area and representative images of pimonidazole staining are shown (green; Hoechst nuclear staining, blue). Scale bar = 1 mm. **e** TC-1 tumor-bearing C57BL/6 mice (*n* = 6/group) were vaccinated with E7 or control (OVA) RNA-LPX at a mean tumor volume of 165 mm^3^ and locally irradiated with 12 Gy. Four days after irradiation, mice were injected with 1 mg BrdU base-analog followed by organ excision on the next day. The fraction of BrdU^+^ tumor-infiltrating CD4^+^ and CD8^+^ T cells is shown. Data are shown as mean ± SEM. Significance was determined using **a**-**d** one-way ANOVA and Tukey’s multiple comparison test and **e** unpaired, two-tailed Student’s t-test with *p ≤ 0.05, **p ≤ 0.01, ***p ≤ 0.001. BrdU: bromodeoxyuridine; LRT: local radiotherapy; LPX: lipoplex; MHC: major histocompatibility complex; MFI: median fluorescence intensity; PD-L1: programmed death ligand 1

As a cytotoxic therapy, LRT potently induced a threefold reduction of TC-1 tumor cell counts (tumor cells/mg tumor, Fig. 3a), and increased the fraction of apoptotic tumor cells, characterized by the expression of CC3 (Fig. 3b) and the death receptor Fas (Fig. 3c), when compared to control or E7 RNA-LPX vaccinated mice alone. However, the expression of MHC class I, *T *cell inhibitory ligand PD-L1, and the inhibitory immune receptor Qa-1^b^ (Fig. 3c) were only mildly modulated by LRT. Conversely, E7 RNA-LPX vaccination strongly increased the expression of MHC class I molecules, PD-L1 and, to a lesser extent, Qa-1^b^ (Fig. 3c) on tumor cells, despite not changing the total tumor cell count (Fig. 3a) and only slightly increasing CC3 and Fas expression on tumor cells (Fig. 3b, c) when compared to control RNA-LPX. TC-1 tumors of combination therapy-treated mice shared features of both monotherapies in which LRT-mediated cell death (Fig. 3a, b) was paired with E7 RNA-LPX-mediated induction of MHC class I and PD-L1 expression (Fig. 3c); however, expression of the cell death receptor Fas and *T *cell inhibitory ligand PD-L1 exceeded those of either monotherapy (Fig. 3c).

Previous in vitro studies using human tumor cell lines have shown that sublethal irradiation can render tumor cells more susceptible to antigen-specific CD8^+^ *T *cells, upregulating the expression of cell surface proteins involved in *T *cell recognition such as Fas, intercellular adhesion molecule 1 and MHC class I molecules [31]. To identify if LRT sensitizes TC-1 tumors to E7-specific CD8^+^ *T *cell-induced death, we co-cultured differentially irradiated TC-1 tumor cells (0 to 12 Gy) with CD8^+^ *T *cells isolated from spleens of E7 RNA-LPX-vaccinated mice in a controlled manner in vitro and measured the expression of MHC class I, PD-L1 and CC3 on tumor cells and the secretion of effector cytokines by E7-specific CD8^+^ *T *cells (Supplementary Fig. 3). Reflecting the previous in vivo observation, co-culture with E7-specific CD8^+^ *T *cells strongly increased the expression of MHC class I and PD-L1 on tumor cells (Supplementary Fig. 3b, c), likely a feedback mechanism in response to IFNγ secretion [32], whereas expression levels remained the same whether tumor cells were irradiated or not. In line with previous reports [31, 33, 34], radiation appeared to sensitize TC-1 tumor cells to E7-specific CD8^+^ *T *cell killing with a higher fraction of cells being CC3^+^ (Supplementary Fig. 3d). In addition, E7-specific CD8^+^ *T *cells secreted more IFNγ when co-cultured with irradiated tumor cells (Supplementary Fig. 3e), suggesting that they may have enhanced recognition of irradiated versus non-irradiated TC-1 tumor cells. These in vitro data support the hypothesis that radiation renders tumor cells more susceptible to antigen-specific CD8^+^ *T *cell-mediated killing. The molecular mechanisms that drive radiation susceptibility are likely multifactorial and were not further evaluated.

In addition to radiation-induced reduction of tumor cell count and increase of tumor cell killing as compared to control RNA-LPX and E7 RNA-LPX alone (Fig. 3a, b), we characterized radiation-mediated effects on the local tumor microenvironment (TME) that could impact intratumoral E7-specific CD8^+^ *T *cells induced by the E7 RNA-LPX vaccine (Fig. 2d). Hypoxia is a hallmark of most solid tumors and commonly promotes immunosuppression [35]. The hypoxia probe pimonidazole [36] was intravenously injected into control, E7 RNA-LPX-, LRT- and combination therapy-treated TC-1 tumor-bearing mice and the hypoxic tumor areas were analyzed by histology (Fig. 3d). In E7 RNA-LPX/LRT-treated mice, TC-1 tumor hypoxia was significantly reduced compared to all other treatment groups (Fig. 3d). The level of tumor hypoxia thereby correlated with the tumor size at the time point of excision (Supplementary Fig. 4) and tumors of E7 RNA-LPX/LRT-treated mice shared the same hypoxic area than untreated TC-1 tumors at matched tumor sizes (explanted at an earlier time point, day 16). Despite similar levels of tumor oxygenation, vascularization was slightly reduced in combination therapy-treated mice (reveled by CD31 staining of endothelial cells), whereas vasculature architecture was similar (using FITC-dextran vessel leakiness assay) between rejecting E7 RNA-LPX/LRT (day 26) and control tumors (day 16) at matched tumor sizes (data not shown), indicating that neither tumor size nor normalization of the vessel phenotype/morphology seem to play the exclusive role in tumor oxygenation observed after combined E7 RNA-LPX/LRT.

The reduction of tumor hypoxia in E7 RNA-LPX/LRT-treated mice was furthermore was associated with markedly increased proliferation of CD8^+^ tumor infiltrated lymphocytes (TIL) five days after LRT (Fig. 3e), as shown by the higher incorporation of the base analog BrdU in CD8^+^ TIL but not in CD4^+^ TIL.

### ***LRT prolongs the duration of E7-specific CD8***^+^*T *cell*** immune responses***

**Fig. 4:**
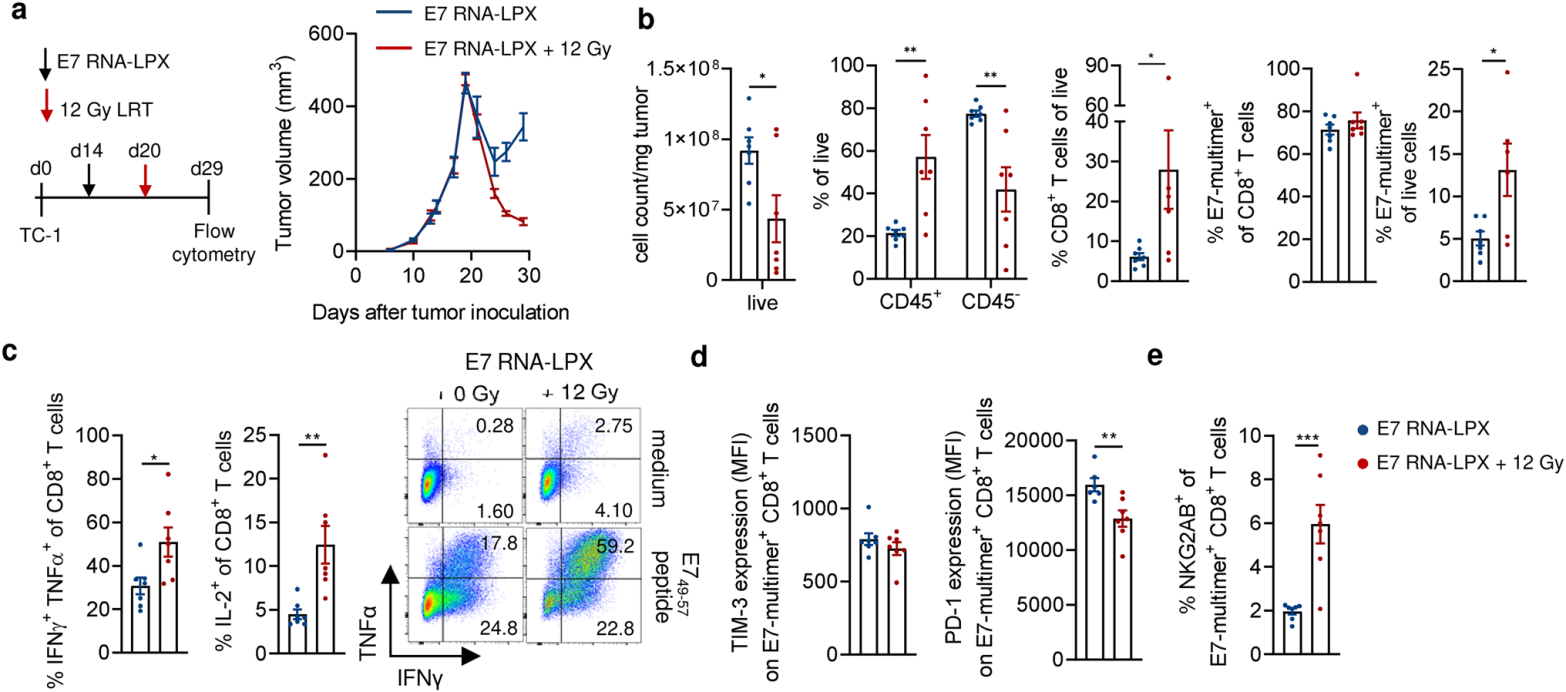
LRT prolongs the duration of E7-specific CD8^+^ T cell immune responses. **a** TC-1 tumor-bearing C57BL/6 mice (*n* = 7/group) were vaccinated with E7 RNA-LPX at a mean volume of 120 mm^3^ and subsequently locally irradiated with 12 Gy. Tumor volume monitored over time. Nine days after irradiation, tumors were excised and characterized via flow cytometry. **b** Fraction of live cells per mg of tumor, CD45^+^ and CD45^−^ cells, CD8^+^ T cells of live, E7-multimer^+^ of CD8^+^ T cells and of live in TC-1 tumors. **c** Production of IFNγ, TNFα and IL-2 cytokines after ex vivo restimulation of enriched CD8^+^ TIL with E7_49–57_ peptide-loaded C57BL/6 BMDC. Representative pseudocolor plot of TNFα and IFNγ expression in CD8^+^ TIL. **d** Expression of TIM-3, PD-1 and **e** the fraction of NKG2AB^+^ cells in E7-specific CD8^+^ TIL. Data are shown as mean ± SEM. Significance was determined using unpaired two-tailed Student’s *t*-test **b**-**e** with *p ≤ 0.05, **p ≤ 0.01, ***p ≤ 0.001. IFN γ: interferon γ; IL-2: interleukin-2; LPX: lipoplex; LRT: local radiotherapy; MFI: median fluorescence intensity; PD-1: programmed death 1; TIL: tumor infiltrated lymphocytes; TIM-3: T cell immunoglobulin and mucin domain-3; TNF α: tumor necrosis factor α

Although we did not observe differences in the E7-specific CD8^+^ *T *cell response in E7 RNA-LPX and E7 RNA-LPX/LRT-treated mice five days after LRT (Fig. 2d, e), the reduction of tumor cell count, tumor hypoxia and increased CD8^+^ TIL proliferation led us to investigate the antigen-specific CD8^+^ *T *cell response when tumor growth curves diverged more strongly, namely day 29 after tumor injection (Fig. 4a) and hypotheized that a more oxygenated environment can have subsequent effects on immune cell types, such as *T *cells. Therefore, we characterized functional parameters of E7-specific CD8^+^ *T *cell responses such as total tumor infiltration, the secretion of cytokines involved in *T *cell effector function (IFNγ, TNFα) and proliferation (IL-2), as well as the expression of negative immune checkpoints (TIM-3, PD-1 and NKG2AB [a Qa-1b ligand [37]) (Fig. 4). Flow cytometry analysis showed that E7 RNA-LPX/LRT treatment induces cell death of tumor cells and hence increases the tumor infiltration of total CD45^+^ cells and CD8^+^ *T *cells, but does not change the fraction of vaccine-induced E7-specific CD8^+^ *T *cells among CD8^+^ *T *cells, when compared to E7 RNA-LPX-treated mice (Fig. 4b). E7-specific CD8^+^ TILs from combination treated mice produced significantly more of the effector cytokines IFNγ and TNFα, as well as IL-2, upon in vitro antigen-specific restimulation (Fig. 4c), indicating that combined LRT treatment drives a higher effector function and activation status of vaccine-induced E7-specific CD8^+^ *T *cells. E7-specific CD8^+^ *T *cells of E7 RNA-LPX/LRT-treated tumors further displayed comparable expression of the immune checkpoint inhibitory receptors TIM-3, a lower expression of PD-1 (Fig. 4d) and a higher expression of the *T *cell inhibitory receptor NKG2AB (Fig. 4e) than E7-RNA-LPX treated mice. The latter likely correlates with the enhanced IFNγ secretion observed in the combination treated group (Fig. 4c) as NKG2AB is known to be expressed after continuous IFNγ secretion in the TME [37].

The higher magnitude and effector function of E7-specific CD8^+^ TILs in E7 RNA-LPX/LRT-treated mice is in agreement with more potent anti-tumor effects observed in vivo.

Together, our data indicate that LRT reduces tumor cell count and tumor hypoxia, thereby amplifying vaccine-induced E7-specific CD8^+^ TIL effector function and promoting rejection of HPV16^+^ tumors.

## Discussion

We herein propose a novel combination therapy regimen for the treatment of advanced HPV16^+^ malignant disease, utilizing standard-of-care LRT in conjunction with an RNA-LPX-based HPV16 vaccine. Our data suggest superior therapeutic efficacy of combining E7 RNA-LPX vaccine with cytotoxic radiotherapy in two HPV16^+^ mouse tumor models when compared to monotherapies.

Based on our observations, E7 RNA-LPX vaccination rendered poorly immune-infiltrated and cold TC-1 tumors immunologically hot, whereas LRT-mediated tumor cell death, reduced tumor cell count and diminished intratumoral hypoxia, which in turn appeared to result in more potent and durable vaccine-primed E7-specific CD8^+^ *T *cell responses during later treatment time points. Restored tumor oxygenation in E7 RNA-LPX/LRT-treated mice did not seem to simply be a result of reduced tumor size, however, further analysis (*e.g.* vasculature morphology and phenotype) would be necessary to fully understand the result of higher oxygen levels and how this influencuences secondary effects such as more durable cellular immune responses.

To date, various studies have evaluated the therapeutic efficacy of DC-[38], DNA-[18, 19], peptide-[20–22] and RNA-based [13] HPV vaccines alone and in combination with LRT [18, 19, 21, 34] in the same tumor models used in this study. LRT was required to potentiate vaccine-induced E7-specific CD8^+^ *T *cell responses in these studies [18, 19, 21], which is in contrast to findings made here, where the systemically administered and inherently innate immune-stimulatory E7 RNA-LPX vaccine [13] is fully capable of priming high numbers of cytotoxic E7-specific CD8^+^ *T *cells. In our hands, differently from most of the previous studies using HPV vaccine-LRT combinations, combining E7 RNA-LPX with LRT timidly modulated the E7 RNA-LPX-primed E7-specific CD8^+^ *T *cell response in magnitude, but rather boosted and sustained their anti-tumor efficacy and effector function at later time points during tumor rejection. In other words, LRT-induced cell death and hence, a reduction of immunosuppressive factors such as tumor load and hypoxia, allows proliferation and prolongs the cytotoxic effector function of tumor-infiltrated E7-specific CD8^+^ *T *cells.

Of notice, among the different vaccine platforms evaluated, the RNA-LPX systemically injected (i) does not require adjuvant components to initiate an antigen specific immune response, (ii) does not integrate into the host genome and (iii) displays the advantage of targeting several lymphoid organs, mainly the spleen with a high reservoir of APC. The high number of APC resident in the spleen provide vaccine-derived antigens to *T *cells, which acquire a broad homing capacity, able to infiltrate in many tissues, as we described in several studies [13, 14, 39]. For certain locally injected peptide- or DNA-based vaccines (*e.g.*, intramuscularly, intranasally or subcutaneously) on the contrary, the *T *cell priming would be restricted to the draining lymph node with much lower number of APCs [40] and with a homing imprinting, perhaps limiting the trafficking of primed *T *cells to certain tissues [41–43].

In E7 RNA-LPX/LRT-treated mice, total myeloid cells, DC1, DC2, M1 TAM, but also suppressive MDSC were enriched. The strong reduction of alternatively activated, inhibitory M2-polarized TAM paired with a high frequency of DC2, M1-polarized TAM and activation of these cells (following the expression of CD86 and MHC class II), suggested that the anti-tumoral effect of combination therapy was supported or resulted in the infiltration and polarization of inflammatory rather than suppressive myeloid cells. However, the level of PD-L1 also increased on several myeloid cell subsets, which likely is a feedback mechanism to the increased inflammation observed. Upon vaccination with RNA- and peptide-based HPV vaccines, we and others previously observed a polarization of TAM toward M1 [13, 43] that, in the context of combined LRT, was further increased. Of notice, MDSC cells, despite known to confer radioresistance [44], have also recently been described for their anti-tumoral role in sustaining immunotherapy (*e.g.*, the HPV peptide vaccine) [43].

Elevated expression of the transcription factor TOX1, which is known to initiate and sustain cytotoxic *T *cell function in cancer and chronic viral infection under prolonged antigen presentation, [29, 30, 45] was observed on E7-specific CD8^+^ *T *cells from E7 RNA-LPX/LRT-treated mice. This may be due to the fast initiation of cytotoxic activity during tumor rejection. Additionally, despite superior tumor rejection, a higher expression of the of the inhibitory receptor NKG2A, which is frequently elevated in patients with HPV16^+^ cancer [37], was observed on E7-specific CD8^+^ TIL taken from mice treated with combination therapy, indicating that triplet combination therapy with anti-NKG2A immune checkpoint blockade [37] could further boost the anti-tumor efficacy of combined E7 RNA-LPX/LRT in TC-1 tumor-bearing mice and patients with HPV16^+^ cancer.

As clinical treatment protocols (70 Gy in 35 fractions over 7 weeks or for radical radiotherapy 65 Gy in 30 fractions over 6 weeks) [46] would exceed the time frame of standard mouse experiments, [24] higher single doses are commonly used in preclinical studies, [33, 47, 48] rather resembling palliative schedules (8 Gy single fraction, 20 Gy in 5 fractions over 1 week, 30 Gy in 10 fractions over two weeks or 27 Gy in 6 fractions) [46], *e.g*., 12 Gy or the similar BED of 3 × 6 Gy LRT used in this study. Preclinical evidence suggests that high-dose-per-fraction LRT is particularly able to prime in situ *T *cell responses [49] which would argue for a different biological effect of combined E7 RNA-LPX/LRT depending on whether E7 RNA-LPX is combined with 2 Gy or 12 Gy fractionated LRT. Given that LRT played a more cytotoxic role within this study, we conclude that total dose rather than dose-fractionation was decisive for the observed synergistic effects. The limitation, however, remains that preclinical radiation schedules do not resemble clinical radiation schedules and there is a chance that combination therapy mediated effects of radioimmunotherapy observed in preclinical studies differ in a clinical situation.

Recent reports have shown that LRT-mediated innate immune infiltration and in situ *T *cell priming is a result of cytoplasmic DNA accumulation and activation of the cyclic GMP-AMP synthase (cGAS)/stimulator of interferon genes (STING) pathway [50, 51]. DNA viruses, such as HPV, antagonize cytoplasmic DNA sensing by cGAS/STING through the inhibition of STING by E7 [52] which could explain the low infiltration of immune cells in HPV16 E6/E7^+^ TC-1 tumors after LRT.

We previously showed that vaccination with RNA-LPX encoding for CD4^+^ *T *cell neoantigens augments cellular responses generated by LRT via in situ release of tumor antigens in a mouse tumor model of colorectal carcinoma, CT26 [25]. With the data presented here, we add to the repertoire of possible mechanisms of combined cancer vaccination and LRT, which may deviate in dependence on multiple factors such as tumor innate immune infiltration, innate immune sensing, pre-existing adaptive immune responses, tumor-mutational burden, CD4^+^ or CD8^+^ *T *cell reactive neoantigens, radiosensitivity and tumor immune suppression.

The safety and therapeutic efficacy of an HPV16 E6/E7 RNA-LPX vaccine is currently investigated in a phase I clinical trial in patients with HPV-driven cancers including HNSCC, anogenital, cervical and penile cancers (NCT03418480) and in a phase II clinical trial in combination with pembrolizumab in patients with HPV16^+^ and PD-L1^+^ HNSCC (NCT04534205). The data of combined E7 RNA-LPX vaccination and LRT in mouse tumor models presented here thus provide a timely first insight into how and what should be considered for such combinations in a clinical application. Combined E7 RNA-LPX/LRT treatment could be suited for patients not eligible for chemotherapy, in a pre-irradiation or in a palliative setting.

### Supplementary Information

Below is the link to the electronic supplementary material.

Supplementary file1 (PDF 446 KB)

## Data Availability

All data relevant to the study are included in the article or uploaded as supplementary information.

## Abbreviations

ANOVA: Analysis of variance
APC: Antigen presenting cells
BED: Biologically effective dose
BMDC: Bone marrow-derived dendritic cells
BrdU: Bromodeoxyuridine
CC3: Cleaved caspase-3
cGAS: Cyclic GMP-AMP synthetase
CR: Complete response
DC: Dendritic cells
DNA: Deoxyribonucleic acid
HNSCC: Head and neck squamous cell carcinoma
HPV: Human papilloma virus
IFNγ: Interferon γ
IL-2: Interleukin-2
LRT: Local radiotherapy
MHC: Major histocompatibility complex
MDSC: Myeloid derived suppressor cells
NK: Natural killer
OVA: Chicken ovalbumin
PD-1: Programmed death 1
PD-L1: Programmed death ligand 1
RNA: Ribonucleic acid
RNA-LPX: RNA-lipoplex
s.c.: Subcutaneous
SEM: Standard error of the mean
STING: Stimulator of interferon genes
TAM: Tumor associated macrophages
TIL: Tumor-infiltrating lymphocytes
TIM-3: *T* cell immunoglobulin and mucin domain-3
TMB: Tumor-mutational burden
TME: Tumor microenvironment
TNFα: Tumor necrosis factor α
Treg: Regulatory *T* cell
