# Supplementary material for: Local radiotherapy and E7 RNA-LPX vaccination show enhanced therapeutic efficacy in preclinical models of HPV16+ cancer
Source: Cancer Immunol Immunother. 2021 Dec 31;71(8):1975–88. doi: 10.1007/s00262-021-03134-9 (PMC9293862; doi:10.1007/s00262-021-03134-9)
Supplement: Supplementary file 1 — Supplementary file1 (PDF 446 KB) [file 262_2021_3134_MOESM1_ESM.pdf]

Supplementary Figure 1

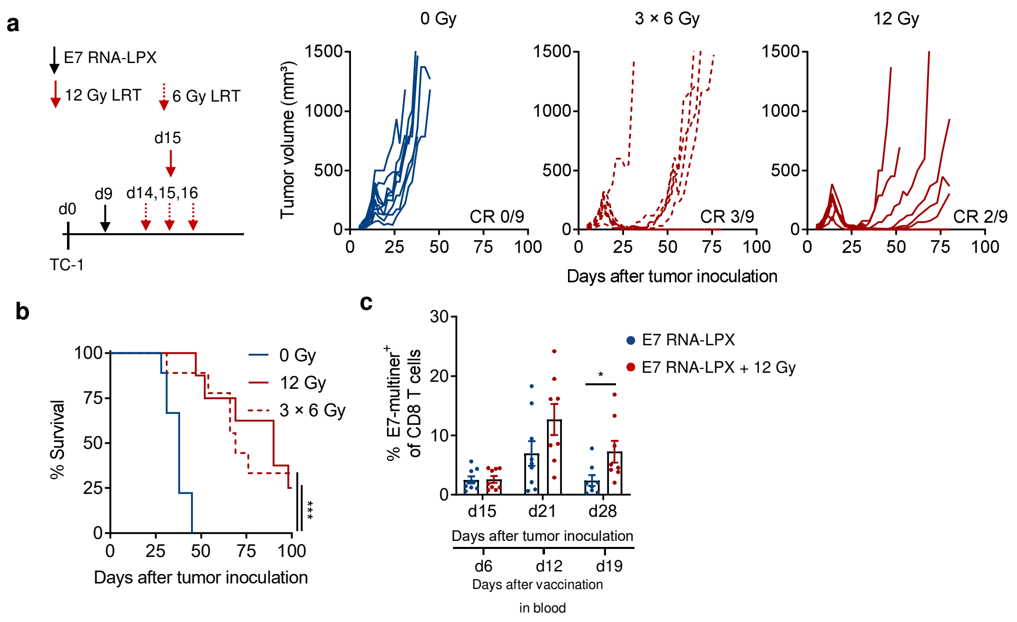

Supplementary Figure 1: E7 RNA-LPX/LRT-mediated anti-tumor effects are not dependent on LRT dose-fractionation.

(a) TC-1 tumor-bearing C57BL/6 mice (n=9/group) were immunized with E7 RNA-LPX at a mean tumor volume of 50 mm^3 and locally irradiated with 12 Gy or a similar BED of 3 × 6 Gy. Individual tumor growth and (b) survival curves are shown. (c) Fraction of E7-multimer$^+$ CD8$^+$ T cells in the blood of TC-1 tumor-bearing mice at different time points. Significance was determined using the Mantel-Cox log-rank test (b) and one-way ANOVA and Dunnett's multiple comparison test (c) with *p ≤ 0.05 for (b) CR: complete response; LPX: lipoplex; LRT: local radiotherapy.

Supplementary Figure 2

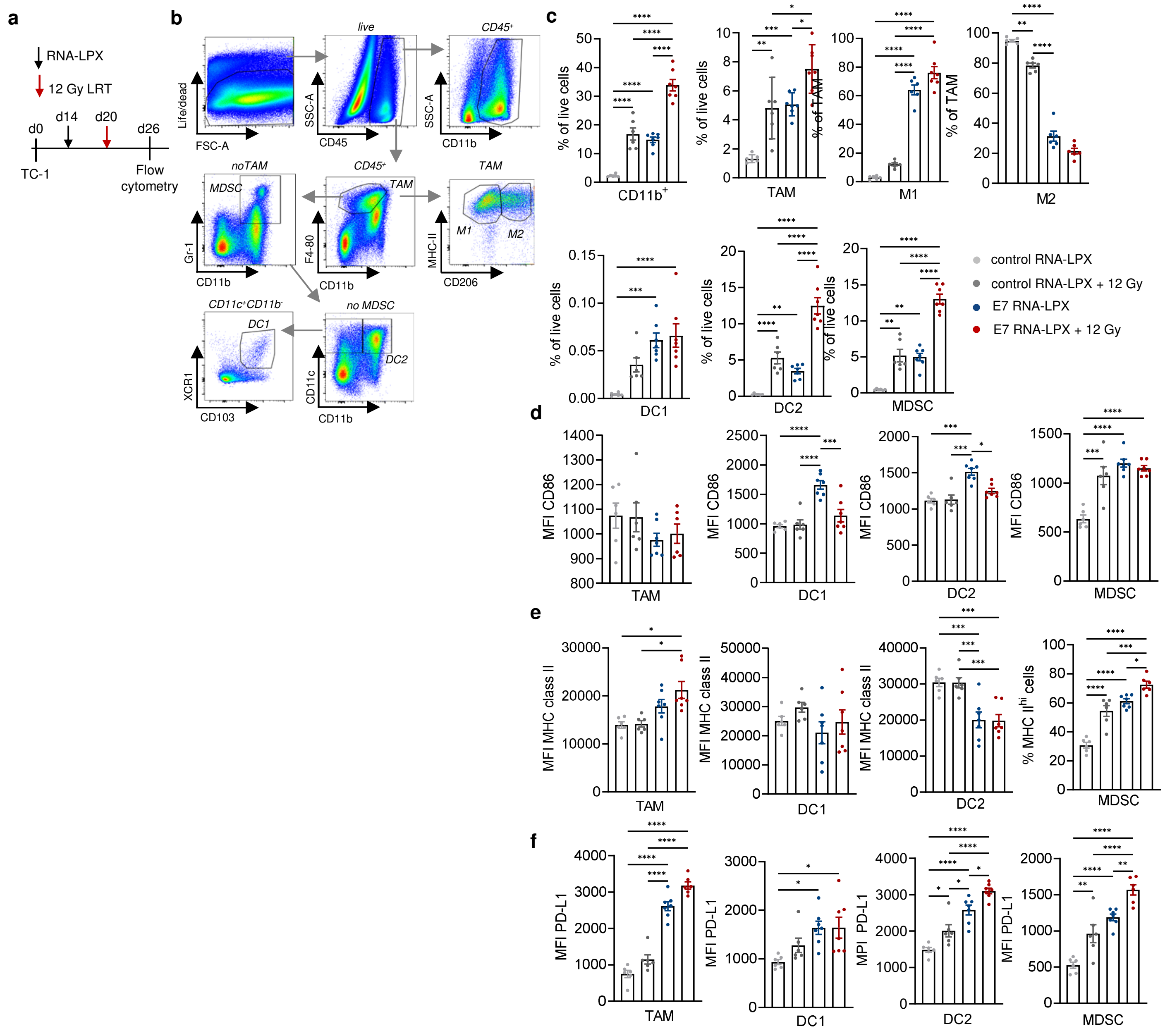

Supplementary Figure 2: E7 RNA-LPX vaccination in combination with LRT increases intratumoral infiltration of myeloid cells.

(a) TC-1 tumor-bearing C57BL/6 mice (n=5/group) were vaccinated with E7 RNA-LPX or control (OVA) RNA-LPX at a mean tumor volume of 120 mm^3 and locally irradiated with 12 Gy. Cellular composition evaluated by flow cytometry after excision of tumors six days after irradiation. (b) Gate strategy used to identify the myeloid cell subsets. Percentage of tumor-infiltrating CD11b$^+$ cells, TAM, M1 and M2 TAM, DC1, DC2, MDSC and expression of activation markers (c) CD86, (d) MHC class II and (e) PD-L1 on indicated populations. Data are shown as mean±SEM. Significance was determined using (b-e) one-way ANOVA and Tukey's multiple comparison test with *p ≤ 0.05, **p ≤ 0.01, ***p ≤ 0.001, ****p ≤ 0.0001. TAM: tumor associated macrophages, MDSC: myeloid derived suppressor cells; DC: dendritic cells, DC1: type 1 DC, DC2: type 2 DC.

Supplementary Figure 3

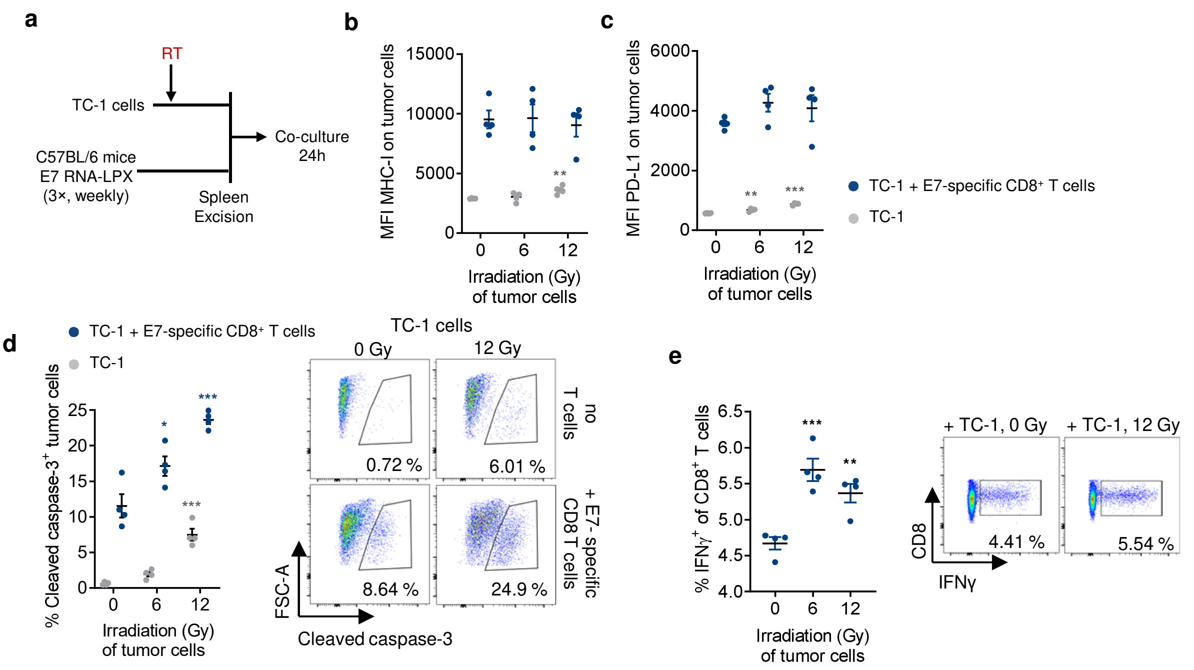

Supplementary Figure 3: Radiation sensitizes cultured HPV16 E6/E7⁺ TC-1 tumor cells to E7-specific CD8⁺ T cell killing.

(a) Irradiated TC-1 tumor cells (0–12 Gy) were co-cultured with E7-specific CD8⁺ T cells (n=4 replicates/condition). C57BL/6 mice were immunized thrice with E7 RNA-LPX, and CD8⁺ T cells enriched from total splenocytes. Tumor cells were irradiated 24 h prior to T cell co-culture. After 24 hours of co-culture, cells were evaluate by flow cytometry. Expression of (b) MHC class I (pan H-2), (c) PD-L1 and (d) CC3 in irradiated TC-1 tumor cells. (e) Fraction of IFNγ⁺ CD8⁺ T cells after CD8⁺ T cell restimulation with irradiated TC-1 tumor cells as determined by intracellular cytokine staining. Representative pseudocolor plots show CC3 staining in TC-1 tumor cells (d, right) and IFNγ staining in CD8⁺ T cells (e, right). Data are shown as mean±SEM. Significance was determined using (b–e) one-way ANOVA and Dunnett's multiple comparison test, comparing the means of all groups to the non-irradiated control group (0 Gy) and (b–d) comparing TC-1 tumor cells alone (grey) to TC-1 tumor cells cocultured with- E7-specific CD8⁺ T cells (blue) with *p ≤ 0.05, **p ≤ 0.01, ***p ≤ 0.001. IFNγ: interferon γ; LPX: lipoplex; MHC: major histocompatibility complex; PD-L1: programmed death ligand-1; RT: radiotherapy.

Supplementary Figure 4

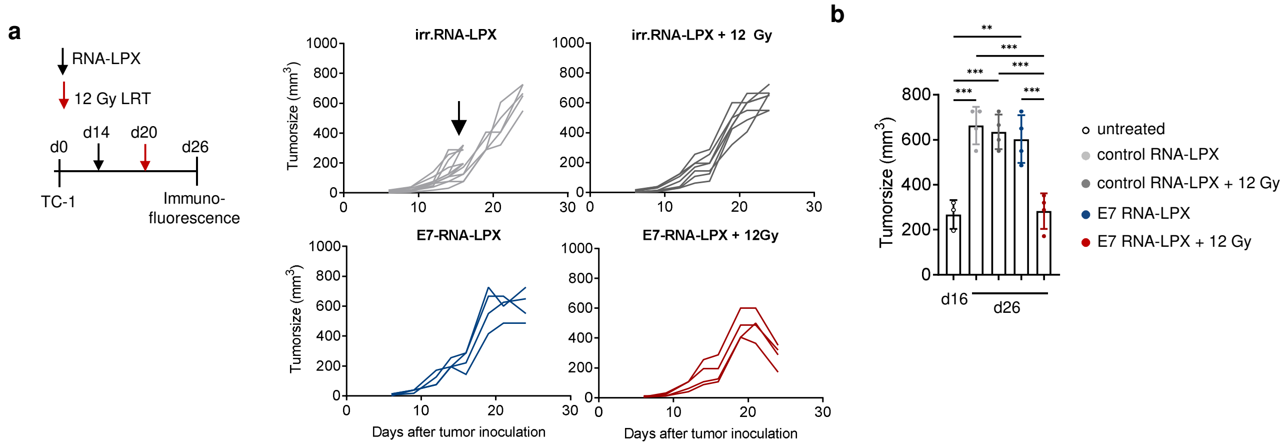

Supplementary Figure 4: Combining LRT with E7 RNA-LPX vaccination reduces intratumoral hypoxia (a) TC-1 tumor-bearing C57BL/6 mice (n=4/group) were immunized with E7 RNA-LPX or control (OVA) RNA-LPX at a mean tumor volume of 160 mm^3 and locally irradiated with 12 Gy as described in Figure 3d . (b) TC-1 tumor size of the mice injected with the hypoxia probe pimonidazole on day 16 (untreated, arrow) and on day 26. Significance was determined using the one-way ANOVA and Turkey's multiple comparison test with *p ≤ 0.05, **p ≤ 0.01, ***p ≤ 0.001. CR: complete response; LPX: lipoplex; LRT: local radiotherapy.
